# High Efficiency Hydrodynamic DNA Fragmentation in a Bubbling System

**DOI:** 10.1038/srep40745

**Published:** 2017-01-18

**Authors:** Lanhui Li, Mingliang Jin, Chenglong Sun, Xiaoxue Wang, Shuting Xie, Guofu Zhou, Albert van den Berg, Jan C. T. Eijkel, Lingling Shui

**Affiliations:** 1 Institute of Electronic Paper Displays, South China Academy for Advanced Optoelectronics, South China Normal University, Guangzhou Higher Education Mega Center, Guangzhou 510006, China; 2 Joint International Research Laboratory of Optical Information of the Chinese Ministry of Education, South China Normal University, Guangzhou Higher Education Mega Center, Guangzhou 510006, China; 3 South China Sea Institute of Oceanology, Chinese Academy of Sciences, Guangzhou 510301, China; 4 BIOS/Lab-on-a-Chip Group, MESA+Institute for Nanotechnology, University of Twente, Postbox 217, 7500 AE Enschede, the Netherlands

## Abstract

DNA fragmentation down to a precise fragment size is important for biomedical applications, disease determination, gene therapy and shotgun sequencing. In this work, a cheap, easy to operate and high efficiency DNA fragmentation method is demonstrated based on hydrodynamic shearing in a bubbling system. We expect that hydrodynamic forces generated during the bubbling process shear the DNA molecules, extending and breaking them at the points where shearing forces are larger than the strength of the phosphate backbone. Factors of applied pressure, bubbling time and temperature have been investigated. Genomic DNA could be fragmented down to controllable 1–10 Kbp fragment lengths with a yield of 75.30–91.60%. We demonstrate that the ends of the genomic DNAs generated from hydrodynamic shearing can be ligated by T4 ligase and the fragmented DNAs can be used as templates for polymerase chain reaction. Therefore, in the bubbling system, DNAs could be hydrodynamically sheared to achieve smaller pieces in dsDNAs available for further processes. It could potentially serve as a DNA sample pretreatment technique in the future.

The size of target DNA fragments is a key parameter for next-generation sequencing (NGS) technology^1^,^2^. DNA has a double helical strand structure storing genetic information^3^. The identity and sequence of the nucleotides (A, T, C, G) defines natural species and individuals, which, consequently, makes DNA sequencing a fundamental research area to extract this genetic information^4^,^5^. To obtain random and size-controlled DNA fragments is a key step in next-generation sequencing sample preparation process and gene expression studies. In molecular diagnostics, sections of genes are screened within a sample using various detection methods, where the target gene hybridizes to a complementary probe molecule. Therefore, DNA fragmentation is an important sample pretreatment step for diagnostic applications where short fragments are required for fast hybridization and high sensitivity target detection.

Methods available for DNA fragmentation include enzymatic digestion^6^,^7^,^8^,^9^, sonication^10^,^11^,^12^, nebulization^13^,^14^ and hydrodynamic shearing^15^,^16^,^17^,^18^,^19^,^20^,^21^. These methods have been widely used to produce DNA fragments for different applications. Each of them has its advantages and disadvantages. Enzymatic digestion using restriction endonucleases to fragment DNA in restriction enzyme cutting sites, which is efficient and precise; however, the resulting fragments are not randomly chopped, and some DNAs with high G-C content or tightly packed DNAs cannot be fully enzymatically digested^12^,^16^. Sonication, nebulization, and hydrodynamic shearing are all physical fragmentation methods. Compared to enzymatic digestion, they are more random and better controlled in size and size distribution^22^. Sonication is an efficient and easy method; however, it can be quite variable and difficult to achieve correct size-distribution, tending to cause breaks within AT-rich regions, and cause damage to DNA molecules^12^,^22^.

In both nebulization and point-sink shearing, DNA molecules are fragmented by hydrodynamic shearing forces. The sheared fragment length depends on tube diameter, shape and in-tube flow velocity^16^,^17^,^21^. Nebulization generates random DNA fragments by forcing a DNA solution through a small orifice of a nebulizer^13^,^14^. It is a fast and reproducible method to fragment DNA molecules to small pieces; however, the resulting DNA fragments have a wide size range, and it also requires a large amount of input DNAs and expensive equipment^22^. Fragmentation based on hydrodynamic shearing in fluidic tubes/channels has been reported, and can produce DNA fragments of short fragment length and narrow size-distribution with less DNA damage^21^,^22^; however, the process needs a complex device and well-trained operators. A high efficiency, good repeatability and low cost technology to achieve precise length DNA fragments is still highly required in the standard DNA sample preparation procedure for routine diagnostic processes.

In biotechnological processes, it has been found that exposure to a bubbling system can induce cell damage or protein denaturation^23^,^24^,^25^. The bubbling process typically include three important processes: bubble formation, bubble movement, and bubble bursting. Shear stresses are generated during the bubbling process, such as resulting from gas-liquid friction, drainage during bubble film change, and hydrodynamic forces during bubble bursting. The timescale for foam (bubble) formation and bursting is typically in the sub-millisecond range^26^, and the shear stress generated in the process of a bubble rising through liquid, foam draining and bubble bursting are respectively estimated to be in the range of 10^0^, 10^-1^ and 10^3^ N/m^2^ assuming air bubble diameter of 0.5 cm and a film thickness of 10 μm in pure water at room temperature^25^. These shear stresses would act on the molecules in the system and affect their behavior.

In this report, we investigated the DNA fragmentation in a bubbling fluidic system. When gas is introduced into the DNA solution via a tube, bubbles form at the orifice, rising up, merging and bursting at the air-liquid surface^27^,^28^. The hydrodynamic forces generated during this process are found to break the DNA molecules into smaller pieces. By controlling gas pressure, bubbling time and temperature, we have obtained size-controllable DNA fragments ranging from 10 to 1 Kbp with a narrow size distribution. Ligation and RAPD-PCR experiments using fragmented genomic DNA samples confirmed the usability of the fragmented DNAs in genome sequencing and gene expressing analysis. We show that this hydrodynamic shearing DNA fragmentation method can fragment different DNA samples to desired lengths. The demonstrated method has several advantages. Except for the setup itself (Fig. 1), there is no other chemical or biological materials involved in this bubbling system. The setup is simple and cheap to construct and operate in a general laboratory. The total cost of our setup is about 60 US dollar, and cost per sample is only from the gas consumption depending on the bubbling pressure and time.

## Results

When N_2_ gas is introduced in the DNA solution via the tube, bubbles are created, rising up to the solution surface and bursting there causing shearing. In this work, we have studied the effects of bubbling time, applied gas pressure and temperature on the average fragmented DNA length.

### Gas Pressure

Long DNA fragments can be sheared and fragmented by the hydrodynamic forces produced in the bubbling process acting on the molecules. We have investigated the applied gas pressure effect on the DNA fragmentation results using the bubbling system. The applied pressure was 0.05, 0.10, 0.15, 0.20, 0.25 and 0.30 MPa, and the bubbling time of 60 min was used to ensure maximal fragmentation at that pressure. Figure 2 shows that the average fragment length varies with applied gas pressure. With the increase of gas pressure, the average fragment length decreases dramatically from more than 10 Kbp down to about 1 Kbp with a narrow distribution (Fig. S1). An average fragment length of 1,090 bp was obtained at 0.30 MPa, which we expect can be further decreased by using a better sealed system allowing higher applied pressures. Each experiment was repeated four times, and as can be seen from Fig. 2 that the bubbling system was stable and showed good repeatability. When the bubbling pressure was increased to >0.3 MPa or using a smaller glass vessel, smaller DNA fragments could be obtained (Fig. S3); however, the vessel sealing cap was broken quickly. Therefore, these data of smaller DNA fragments at higher pressure are not shown here.

### Bubbling time

The probability that a molecule undergoes sufficient extension to be fragmented will also be related to the number of times the molecule is exposed to a shearing field. In the bubbling system, the frequency at which a DNA molecule is sheared depends on the amount of bubbles which can be generated per unit vessel volume. At a fixed gas pressure and tube size, the bubble generation frequency and bubble size is constant; therefore, the bubbling time is the determining factor.

Figure 3 shows the effect of bubbling time on fragmentation. We fixed the gas pressure at different values, and kept bubbling in DNA solution in the Teflon cuvette. A drop of DNA solution (20 μL) was taken from the cuvette every 10 min, and the fragment length was measured in electrophoresis experiments together with the original non-sheared sample for comparison. It clearly shows that the fragment length initially decreases with bubbling time, and reaches a plateau after a period of time. The fragment length at time 0 is not shown in the curve because the genomic DNA molecules are longer than 15 Kbp and cannot be precisely measured using our setup. The plateau implies that DNA molecules cannot be further fragmented, and thus indicates the minimum length that can be achieved at that pressure. The time to reach the plateau (*t*_m_) decreases with increasing applied gas pressure (*P*), as shown in Fig. 3(c). It takes 55, 60, 50, 45 and 40 min to reach the plateau respectively at 0.10, 0.15, 0.20, 0.25 and 0.30 MPa.

### Temperature effect

Temperature affects the conformation of DNA molecules and viscosity of DNA solution^27^. Both effects may influence the bubble formation and DNA fragmentation in our experiments. To investigate this influence, we have carried out experiments at an applied pressure of 0.25 MPa and a bubbling time of 60 min at 5, 10, 20, 30 and 40 °C, as shown in Fig. 4. Temperatures of 5–40 °C were chosen to avoid freezing and denaturation of the DNA, of which single-chain molecules will be generated^29^. As shown in Figs 4 and S2, with increasing temperature, shorter DNA fragments were obtained.

Since viscosity decreases with increasing temperature, bubble formation and bubble rise become easier. In hydrodynamic DNA shearing systems, the fragment length increases with temperature in the same range as investigated here or decreasing the viscosity of the DNA solutions^16^. This suggests that in our system the shearing forces generated during bubble formation and bubble rise do not significantly contribute to the DNA fragmentation effect. Therefore we expect that the bubble bursting might generate the major hydrodynamic forces for DNA fragmentation. On the other hand, the DNA molecular conformation and chain flexibility change with temperature. Flexible DNA molecules can be more easily extended on the bubble film, and exposed to larger shearing forces, which might also play a role in the observed increased DNA fragmentation^14^,^25^.

### Process Yield

**Figure 1:**
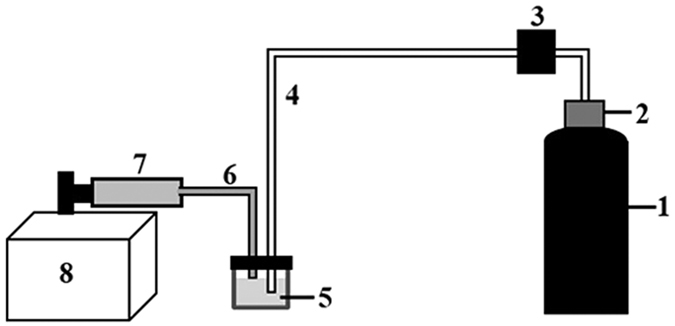
Schematic drawing of the experimental setup. 1 - N_2_ container, 2 - compression release valve, 3 - pressure controller, 4 - Teflon tube, 5 - DNA solution in Teflon cuvette, 6 - buffer supplier tube, 7 - syringe, 8 - syringe pump.

**Figure 2:**
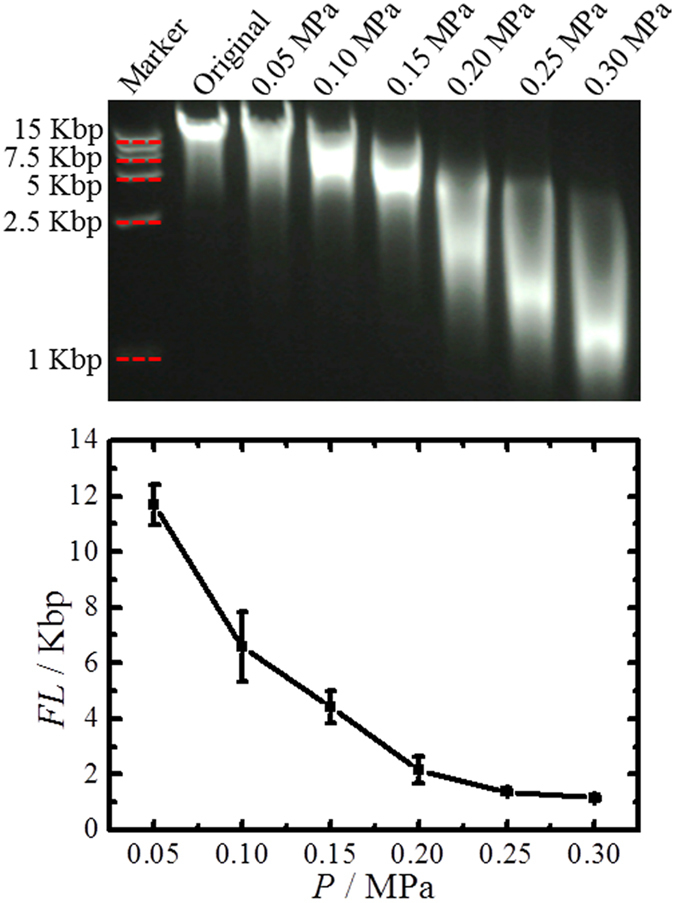
DNA fragment length (*FL*) varies with applied gas pressure (*P*). The standard deviation is calculated from 4 repeated experiments for each point.

**Figure 3:**
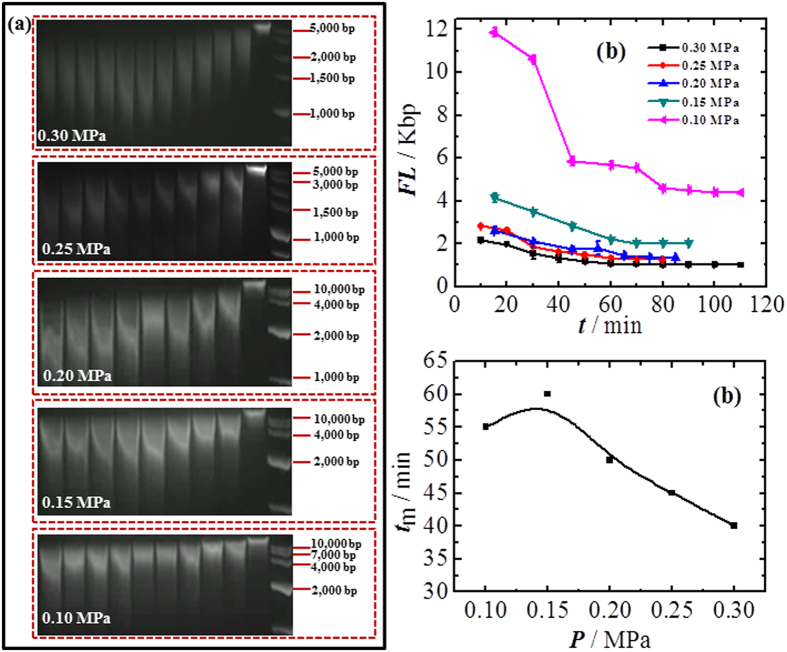
DNA fragment length varies with bubbling time at different gas pressure. **(a)** Gel images of fragmented DNAs obtained at different pressure and time. **(b)** Curve of bubbling time (*t*) versus DNA fragment length. **(c)** Time to reach the plateau of minimum fragment length (*t*_*m*_) varies with applied gas pressure. The standard deviation is calculated from 3 to 4 repeated experiments for each point.

**Figure 4:**
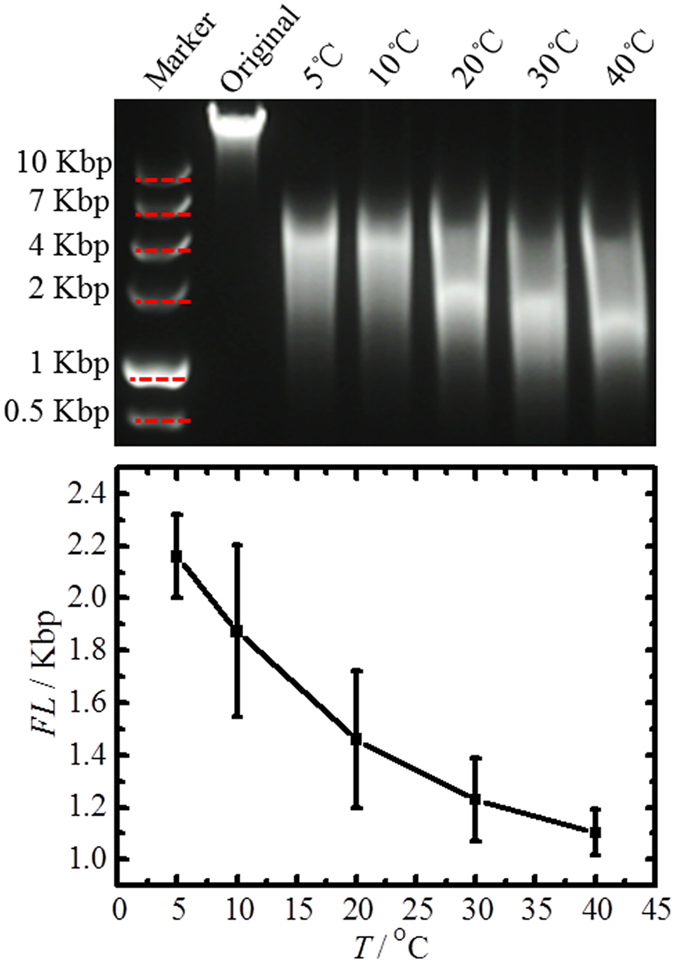
Temperature (*T*) versus average DNA fragment length obtained with applied pressure of 0.25 MPa and bubbling time of 60 min. The standard deviation is calculated from 3 repeated experiments for each point.

Sample loss is a problem for some DNA sample treatment methods^11^,^14^,^19^,^20^,^21^. In this bubbling DNA fragmentation process, water evaporates quickly and DNA sample loss can occur due to sample splashed out and stuck on tube/cuvette surface. Therefore, we designed and applied a continuous buffer supply system to replenish the solution, keeping the sample volume constant, as shown in Fig. 1. A Teflon cuvette has been chosen because of its hydrophobic surface to avoid sample sticking. The yield of the process was calculated by


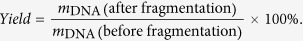


Here *m*_DNA_ is the amount of DNA obtained by multiplying the sample volume with the DNA concentration measured using a microplate reader (Infinite M200 Pro, TECAN, Switzerland).

**Figure 5:**
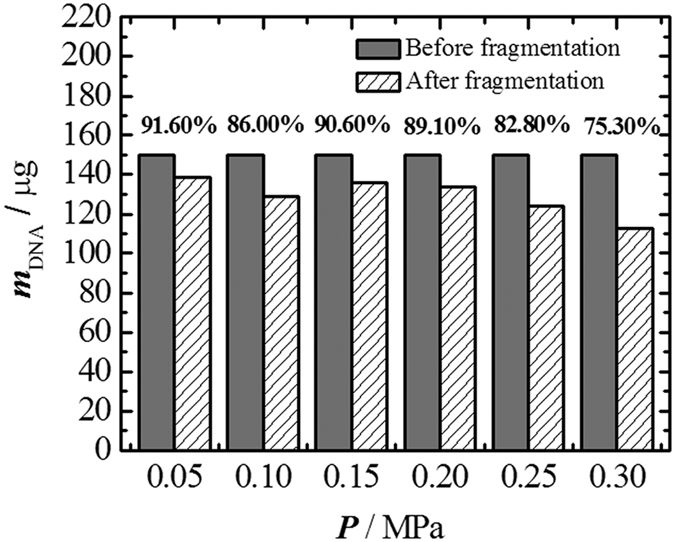
Sample yield sheared under different pressures. The bubbling time is 60 min.

As shown in Fig. 5, the yield varies in the range of 75.30–91.60% at the applied pressures of 0.05–0.30 MPa and bubbling time of 60 min. Higher yield is achieved at lower gas pressure, and we expect it can be further improved with better sealed devices. Typically, a yield of 75% could be guaranteed, which is sufficient for most genomic analysis studies.

### Ligation of Fragmented DNA

The ligation reaction was carried out to check the status of the ends of the fragmented DNAs. Since there are papers reported that hydrodynamic shear and heat may cause double-strand DNA (dsDNA) to denature into single-strand DNA (ssDNA)^30^,^31^. The fragmented DNA samples were first checked by introducing ssDNA-specific nuclease, Exonuclease I (TaKaRa Biotechnology (Dalian) Co., Ltd, China), which can digesting ssDNA but not dsDNA. Results from agarose gel electrophoresis indicate that the fragmented DNA samples were mainly dsDNAs since majority of the DNAs remain intact after the treatment with Exonuclease I (Fig. S4). The ability of Exonnulease I to digest ssDNA was confirmed here. Additionally, DNA concentration was also measured before and after Exonuclease I digestion using a spectrophotometer (Thermo Scientific NanoDrop 2000, USA) (Table S1). After digestion, the DNA concentration is slightly decreased, which is acceptable considering the multiple sample treatment steps. Moreover, the ratio of optical density (OD) at 260 and 280 nm wavelength (OD_260_/OD_280_) is in the range of 1.8–1.9 before digestion, demonstrating that the DNA samples are quite pure without obvious contamination from RNA, protein or phenol; however, the OD_260_/OD_280_ decreased to <1.6 after digestion, likely due to the presence of enzymes in the reaction solution^32^.

**Figure 6:**
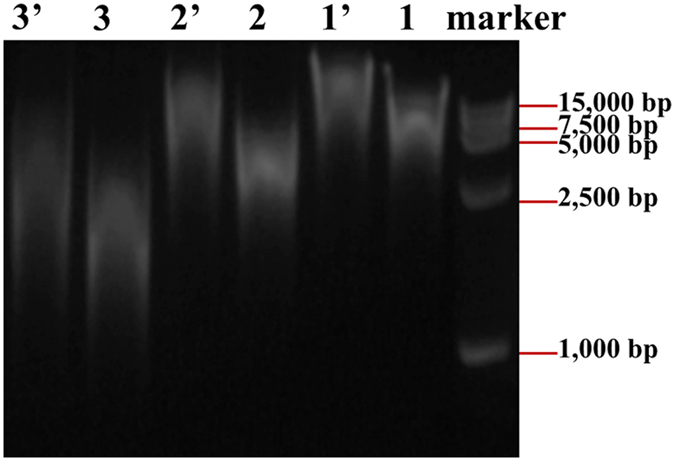
Gel images of the DNAs before and after ligation reaction with 1 and 1′- fragmented genomic DNAs at 0.10 MPa before and after ligation, 2 and 2′- fragmented genomic DNAs at 0.20 MPa before and after ligation, 3 and 3′- fragmented genomic DNAs at 0.30 MPa) before and after ligation. The bubbling time is 60 min for all samples. Molecular weight marker DL 15,000 was used to measure the size of the DNAs.

DNAs sheared under different gas pressure were ligated by T4 DNA ligase in 10 μL ligation reaction mixture, and the ligated DNAs were separated by agarose gel electrophoresis (Fig. 6). Results demonstrate that the length of DNAs after ligation are about twice of their corresponding DNAs before ligation, suggesting that the relatively smaller fragmented DNA molecules are ligated into larger ones. These results show that the ends of the fragmented DNAs are not damaged by hydrodynamic shearing and they are usable for next step process.

Furthermore, two genomic DNA samples (DNAs from Salmon Testes and DNAs from Herring Testes) and one Lambda DNAs with known A-T rich regions^33^ were hydrodynamically sheared using this method (Fig. S5). Gel images with smear bands were obtained.

### RAPD-PCR

**Figure 7:**
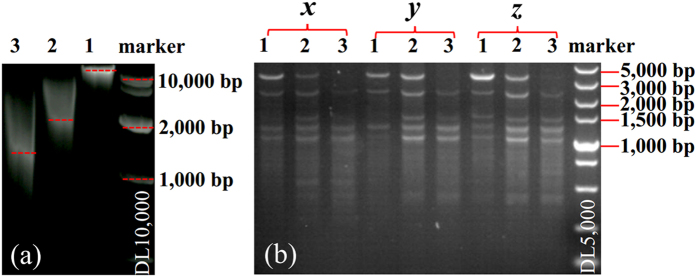
RAPD-PCR experimental results. **(a)** DNA samples used for RAPD-PCR experiments. 1 - untreated genomic DNAs, positive control; 2 - fragmented genomic DNAs (30 min at 0.20 MPa), 3 - fragmented genomic DNAs (30 min at 0.30 MPa). Molecular weight marker DL 10,000 was used to estimate the size of the genomic DNAs. **(b)** RAPD-PCR products of sample 1, 2, 3 in three different reactions using different sets of primers - (*x, y, z*), and molecular weight marker DL 5,000 was used to estimate the size of the PCR products.

Amplification of random regions of genomic DNA using 10-base primers in the random-amplified polymorphic DNA polymerase chain reaction (RAPD-PCR) was used to check the fragmented DNAs as templates for PCR. Three RAPD-PCR reactions were performed for each of the two fragmented DNAs samples (sample No. 2: 30 min at 0.20 MPa; sample No. 3: 30 min at 0.30 MPa). Genomic DNAs without fragmentation were used as positive controls (sample No. 1) (Fig. 7(a)). The average fragment length of sample 1, 2 and 3 are >15 Kbp, ~2.5 Kbp and ~1.2 Kbp, respectively. All three DNA samples can be successfully amplified in three different RAPD-PCR reactions, generating products with specific bands in the range of ~1,000 to 5,000 bp in size (Fig. 7(b)).

For smaller size PCR products (<1.5 kb), the band patterns were very similar for the fragmented and non-fragmented DNA molecules. For larger size PCR products (>3 kb), as expected, weaker bands were observed due to the reduced size of the DNA template in samples 2 and 3. These results suggest that the fragmented genomic DNAs can be used for PCR amplification, and we expect they can also be used for DNA hybridization, mate pair sequencing, shotgun sequencing, genome polymorphism analysis, DNA microarray, paternity test and so on.

In summary, genomic DNAs can be efficiently and unselectively fragmented to dsDNAs by hydrodynamic shearing with fragment ends available for ligation and fragments as templates for PCR. In the future, higher resolution measurements could be obtained to further investigate the capabilities of the technology.

## Discussion

**Figure 8:**
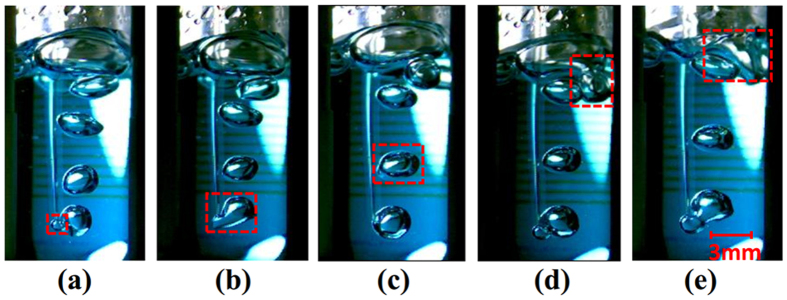
High-speed microscope images of bubbling process. **(a)** Bubble generation, **(b)** bubble dilation and release, **(c)** bubble rise-up, **(d)** bubble coalescence with another one to a larger bubble, **(e)** larger bubble rupture at solution-air interface. This sequence of events is also schematically shown in Fig. 8.

When N_2_ is introduced into the DNA solution via a small tube, bubbles are generated from the tube tip (orifice), and then rise up to the solution surface where they burst to release N_2_ to air and DNA solution back to bulk solution. A high-speed camera (Phantom Miro M110, Vision Research Inc., Wayne County, NC, USA) was used to visualize and record the bubbling process as shown in Fig. 8. The images were taken under a resolution of 1,280 × 800, and the shooting frequency was 1,600 fps. At the pressure of 0.10 MPa, N_2_ gas was released from the orifice of a 0.5 mm inside-diameter tube. A bubble was generated (traced in a red dashed square) (a); grew and released from the orifice (b); rose up to the air/solution interface (c); coalesced with another bubble to form a larger bubble (d); ruptured at the air-solution interface (e).

DNA molecules are commonly coiled in aqueous solution and can be stretched by elongational shear forces^34^. Lentz *et al*. have investigated the mechanism of DNA fragmentation by jet nebulization, and found that hydrodynamic shear is responsible for DNA degradation^13^. Clarkson *et al*. have confirmed that protein denaturation in a foam is correlated with the interfacial exposure leading to conformational changes at the gas-liquid interface^14^,^25^. When the residence time of the DNA fragments in a shear gradient is sufficiently long, it allows extension of the DNA molecules into a highly extended conformation. When the molecule assumes a completely extended configuration the applied forces are greatest and most likely to break covalent bonds. As mentioned before, the bubble film thickness is in the range of a few to tens of micrometers^35^, the timescale for foam (bubble) bursting is in the sub-millisecond range^26^, and the bubble bursting at the air-solution interface generates the highest shear stress during the bubbling process^25^. In this process the bubble film becomes increasingly thinner leading to bubble rupture at the free gas-liquid surface with the thin liquid film breaking up into a number of tiny droplets generating jets of liquid that are projected into the air due to the rush of inflowing gas as the pressure in the bubble is released^28^,^36^.

**Figure 9:**
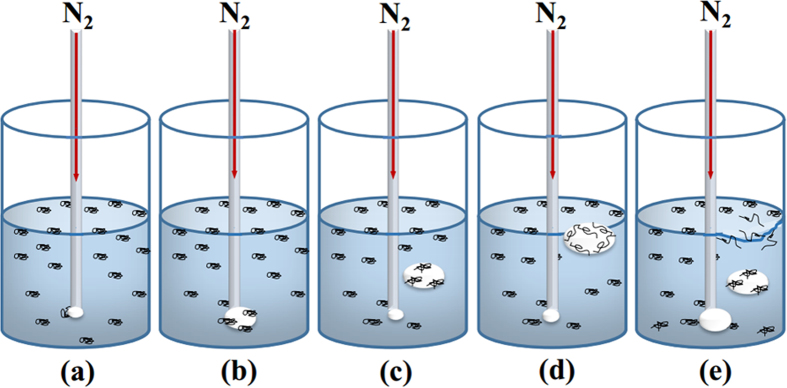
Schematic drawing of DNA fragmentation in the bubbling system. **(a)** Initiation of a gas bubble at the tube orifice, and DNA molecules absorbed to the bubble surface, **(b)** bubble dilation, more DNA molecules to the bubble surface, **(c)** bubble detaching and rising up, DNA molecules move with bubble and sheared by flow around the bubble, **(d)** bubble coalescence, DNA molecules sheared by the extensional strain force, **(e)** bubble bursting, DNA molecules broken by bubble breakup and high-speed liquid jet.

To understand the main contributions to the DNA fragmentation in the bubbling system, several factors have been considered and tested. Firstly, N_2_ was chosen as the gas source of bubbles to avoid oxidation during DNA fragmentation contributing to the process. Secondly, we performed experiments by putting a layer of silicon oil on top of the DNA solution to investigate the effect of bubble bursting on the DNA fragmentation. No obvious DNA fragmentation results were achieved when the DNA solution surface was covered by 1 mL silicon oil, while the same DNA sample could be fragmented to ~8 Kbp without the oil film under the same conditions (bubbling for 30 min at 0.10 MPa, Fig. S6). This points at the bubble bursting and jet formation as the main process causing DNA fragmentation. Furthermore, smaller fragments were obtained at higher temperatures as described above in the Section “Temperature effect”. As mentioned in this section, this also points at the fact that bubble formation and rising are not the main causes of DNA fragmentation. Taking all these factors into account, we conclude that DNA fragmentation in the bubbling system is mainly caused by shearing during the bubble bursting and jet formation (Fig. 9). Multiple fragmentation events will lead to increasingly shorter fragments with the fragmentation continuing until the fragments are too short to be sufficiently extended for shear breaking at different thresholds.

## Materials and Methods

### The bubbling system

The schematic drawing of the bubbling system is show in Fig. 1. Nitrogen gas (99.9999% pure) is released from a N_2_ tank (1) via a pressure release valve (2) and pressure controller (3) through a Teflon tube (4) to the DNA solution in a cuvette (5). To avoid solution loss and to keep the solution volume constant, a syringe pump (Gemini, KD scientific, America) (8) is used to continuously replenish the buffer solvent via a syringe (7) through a Teflon tube (6) to the sample solution (5). All accessories are disinfected in a high-pressure steam sterilizer (TOMY, SX-500, Japan) before using. Gas bubbles are generated and dilated at the tip of the tube 4, then leave the tube to rise up to the air-liquid interface where they burst and release the N_2_ to air. All materials and tools used in our experiments, including Teflon tube, cuvette and connectors were thoroughly cleaned and sterilized before using to minimize external DNA contaminations. Experiments were carried out in a laminar flow clean workbench which had been sterilized using 70% ethanol and ultraviolet light.

### DNA sample preparation

Salmon sperm DNAs (deoxyribonucleic acid sodium salt from salmon testes, CAS: 68938-01-2, Sigma-Aldrich, Shanghai, China), herring sperm DNAs (deoxyribonucleic acid sodium salt from herring testes, CAS: 438545-06-3, Sigma-Aldrich, Shanghai, China), lambda DNAs (duplex DNA, the molecular weight is 31.5 × 10^6^ daltons and it is 48,502 base pairs in length, CAS: B600011, Sangon Biotech, Shanghai, China) were chosen as the DNA samples for experiments. Solid samples were dissolved in sterilized ultra-pure water (>18.25 MΩ cm) in a plastic cuvette at a concentration of 2.50 mg/mL, and stored at −20 °C. When used, the DNA solution was thawed and diluted to a concentration of 100 μg/mL with 0.5 × TE-Buffer (pH = 8.0) (diluted from 5 × TE-Buffer (pH = 8.0) containing 54.00 g/L Tris-base), 27.50 g/L H_3_BO_3_ and 0.001 mol/L Ethylene diamine tetra-acetic acid (EDTA). All other chemicals were purchased from Aladdin Industrial Corporation, Shanghai, China.

### DNA quantification and analysis

DNA solutions and loading buffer of 0.25% bromophenol blue and 40.00% m/v sucrose solution (both purchased from Aladdin Industrial Corporation, Shanghai, China) were loaded onto 100 mL 1.50∼2.00% agarose gels (Sigma-Aldrich, CAS: 9012-36-6, China) containing 5 uL 0.50 mg/mL ethidium bromide. Electrophoretic separation was carried out in a gel electrophoresis apparatus (DYY-8c, Beijing Liuyi Co., Ltd., Beijing, China) at a constant voltage of ∼115 V. Length of the DNA fragments was computed by digitizing agarose slab gels with the Gel Imaging System (BIO-BEST “A” series, SIM, America). The image was calibrated by using marker solutions with known DNA fragment size (all markers used in our experiments were purchased from TaKaRa Biotechnology (Dalian) CO., Ltd, China), and then a cubic spline interpolation function was used to map digitized coordinates to fragment length in base pairs.

### Ligation Reaction

DNAs ligation reaction was performed in 10 μL ligation reaction mixture: 1.2 μg DNA sample, 1 μL T4 DNA Ligase (350 U/μL, T4 DNA Ligase Kit, TaKaRa Biotechnology (Dalian) Co., Ltd, China), 1 μL 10 × T4 DNA Ligase buffer (T4 DNA Ligase Kit, TaKaRa Biotechnology (Dalian) Co., Ltd, China), and sterilized distilled water (added to tune the total volume to 10 μL). The mixture was incubated for 36 h at 15 °C for the ligation reaction. Ligated DNAs were separated by agarose gels with the corresponding fragmented DNAs before and after ligation reaction.

### RAPD-PCR

RAPD-PCR was performed to check the possibility of the fragmented DNAs from the bubbling system as DNA templates for PCR amplification. A microplate reader (Infinite M200 Pro, TECAN, Switzerland) was used to determinate the DNA concentration, and 200 ng of DNAs was used in each RAPD-PCR reaction. Four 10-base RAPD-PCR primers were used, primer 287 (5′-GCAACGGCGG-3′), primer C1 (5′-TTCGAGCCAG-3′), primer K2 (5′-GTCTCCGCAA-3′) and primer F12 (5′-ACGGTACCAG-3′). Primer 287 has been used previously^37^, and all primers 287, C1, K2, F12 were provided by BGI tech (Shen Zhen, China). Three different PCR reactions (*x, y* and *z*) were carried out, respectively. Reaction *x* includes primers 287 and C1, reaction *y* includes primers 287 and K2, and reaction *z* includes primers 287 and F12. The RAPD-PCR reaction was: 95 °C for 5 min followed by 40 cycles of 95 °C for 45 s, 36 °C for 1 min, and 72 °C for 2 min; and 72 °C for an additional 10 min in a PCR Cycler (T100 Thermal Cycler, Bio-Rad, USA).

## Additional Information

**How to cite this article**: Li, L. *et al*. High Efficiency Hydrodynamic DNA Fragmentation in a Bubbling System. *Sci. Rep.* **7**, 40745; doi: 10.1038/srep40745 (2017).

**Publisher's note:** Springer Nature remains neutral with regard to jurisdictional claims in published maps and institutional affiliations.

## Supplementary Material

Supplementary Information
