# Supplementary material for: High Efficiency Hydrodynamic DNA Fragmentation in a Bubbling System
Source: Sci Rep. 2017 Jan 18;7:40745. doi: 10.1038/srep40745 (PMC5241652; doi:10.1038/srep40745)
Supplement: Supplementary Information [file srep40745-s1.pdf]

Supplementary Information

High Efficiency Hydrodynamic DNA Fragmentation in a Bubbling System

Lanhui Li[1,2], Mingliang Jin[1,2], Chenglong Sun[3], Xiaoxue Wang[3], Shuting Xie[1,2], Guofu Zhou[1,2], Albert van den Berg[2,4], Jan C. T. Eijkel[2,4] & Lingling Shui[1,2]*

[1] Institute of Electronic Paper Displays, South China Academy for Advanced Optoelectronics, South China Normal University, Guangzhou Higher Education Mega Center, Guangzhou 510006, China

[2] Joint International Research Laboratory of Optical Information of the Chinese Ministry of Education, South China Normal University, Guangzhou Higher Education Mega Center, Guangzhou 510006, China

[3] South China Sea Institute of Oceanology, Chinese Academy of Sciences, Guangzhou 510301, China

[4] BIOS/Lab-on-a-Chip Group, MESA+ Institute for Nanotechnology, University of Twente, Postbox 217, 7500 AE Enschede, the Netherlands

Email: shuill@m.scnu.edu.cn

Tel.: +86-(0)20-39314813

Fax: +86-(0)20-39314813

Gel image analysis

Agarose gel images that were taken using a gel image system (BIO-BEST "A" series, SIM, America) were analyzed using MATLAB software to quantitate DNA fragment size and size distribution. The plot profile function for each electrophoresis lane is obtained as a curve of gray scale vs. migration distance. The gray scale of the gel image is corresponding to the fluorescence intensity. The size vs. migration distance calibration curve is obtained using the DNA markers (DL15,000 and DL 10,000 DNA markers, TaKaRa Biotechnology (Dalian) CO., Ltd, China). The fragment size of the major peaks is then determined based on the calibration curve of the migration distance. Then the curve of gray scale vs. fragment length (FL) can be obtained. The *FL* indicated on the *x*-axis (Fig. S1 and Fig. S2) is directly mapped using the markers. Figures S1 and S2 are the curves corresponding to Figures 2 and 4.

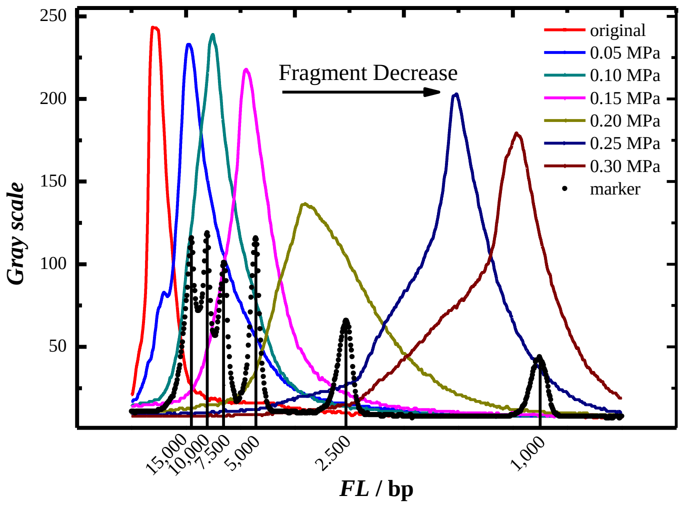

Figure S1. Fragment length distribution. DNA was fragmented at different pressure of 0, 0.05, 0.1, 0.15, 0.2, 0.25, 0.3 MPa for 60 min. DL 15000 that contains fragment length of 15 Kbp, 10 Kbp, 7.5 Kbp, 5 Kbp, 2.5 Kbp and 1 Kbp was used as marker.

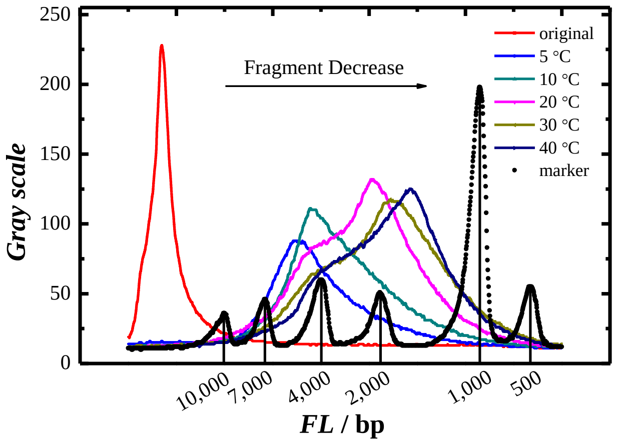

Figure S2. Fragment length distribution. DNA was fragmented at different temperature of 5, 10, 20, 30, 40 °C for 60 min. DL 10,000 that contains fragment length of 10,000 bp, 7,000 bp, 4,000 bp, 2,000 bp, 1,000 bp and 500 bp was used as marker.

DNA fragmentation using a small glass vessel

When the genomic DNAs were hydrodymanically sheared in a smaller glass vessel, as shown in Fig. S4, shorter fragments with *FL* < 1000 bp have been obtained.

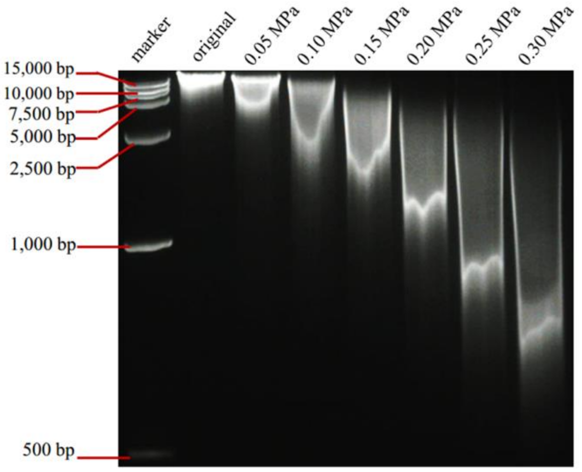

Figure S3. DNA fragment length (*FL*) varies with applied gas pressure (*P*). The shearing was carried out in a glass tube for 20 min.

ssDNA digestion reaction.

The digestion reaction solution contains a 1.2 μg DNA sample (fragmented under different pressure); 6 μL Exonuclease I (5 U/μL, TaKaRa Biotechnology (Dalian) Co., Ltd, China); 2 μL 10×Exonuclease I buffer and sterilized distilled water (to make the total volume up to 20 μL). The mixture was incubated for 30 min at 37 °C. Then 10 μL mixture was taken out to run gel electrophoresis experiments. The ssDNA was purchased from Sigma-Aldrich, Shanghai, China (Deoxyribonucleic acid, single stranded from salmon testes, CAS: 68938-01-2). Figure S4 shows the gel images.

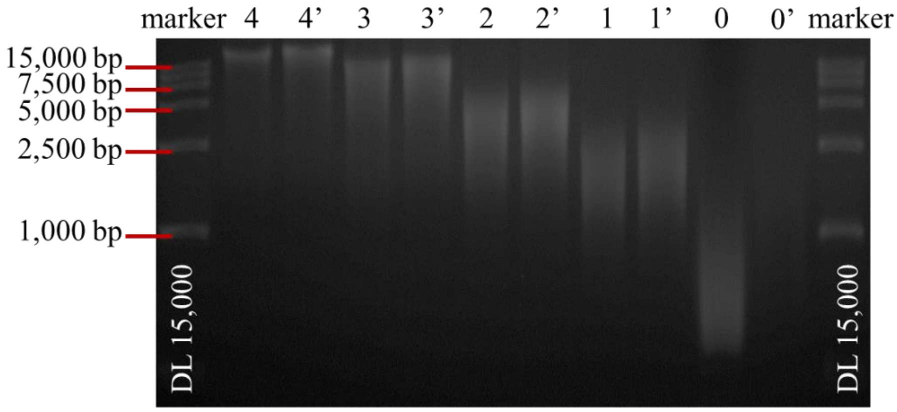

Figure S4. Gel images of ssDNA digestion reaction results: 0 and 0' - ssDNAs before and after digestion, 1 and 1'- fragmented genomic DNAs (sheared at 0.10 MPa, 60 min) before and after digestion, 2 and 2'- fragmented genomic DNAs (sheared at 0.20 MPa, 60 min) before and after digestion, 3 and 3'- fragmented genomic DNAs (sheared at 0.30 MPa, 60 min) before and after digestion，4 and 4' - original DNA samples before and after digestion. The bubbling time is 60 min for all samples. Marker DL 15,000 was used to measure the size of the DNAs.

The concentration of dsDNAs before and after digestion was measured by a spectrophotometer (Thermo Scientific NanoDrop 2000, USA). The results are shown in Table S1. Each data were averaged by 5 measurements.

Table S1 Measurement results from Nanodrop 2000

	Before Digestion		After Digestion	
Pressure MPa	dsDNA concentration (ng/μL)	OD_{260}/OD_{280}	dsDNA concentration ng/μl	OD_{260}/OD_{280}
0.0	82.56	1.86	82.64	1.37
0.1	77.40	1.86	72.01	1.37
0.2	80.10	1.85	72.37	1.5
0.3	78.16	1.84	75.55	1.47

Fragmentation of Herring Sperm DNA and Lambda DNA

Herring Sperm DNAs (deoxyribonucleic acid sodium salt from herring testes, CAS: 438545-06-3, Sigma-Aldrich, Shanghai, China) and Lambda DNAs (48.502 Kbp, deoxyribonucleic acid, bacteriophage (Lambda Phage) from Escherichia coli (No. B600011, Sangon Biotech, Shanghai, China) were hydrodynamically sheared using this method (Fig. S2). Herring Sperm DNA is a kind of natural DNA similar to Salmon Sperm DNA that we used in our experiments. The Lambda DNA is a type of DNA with A-T rich sequences (promoters in genetics) [Talaer, J. Y. L. & Jeanteur, P. Preferential binding of E. coli RNA-polymerase to A—T rich sequences of bacteriophage lambda DNA. *Febs Letters* **12**, 253-256, (1971)]. If the hydrodynamic fragmentation happens easier at A-T region than G-C region, specific DNA patterns should be observed. We carried out an experiment using this Lambda DNA, as shown in Figure S2b. The fragmented DNAs show a smear pattern similar to other two genomic DNAs (Salmon Sperm DNA(Figure 2 and 3) and Herring Sperm DNA(Figure S2(a)). Based on current experimental results we obtained, the hydrodynamic DNA fragmented in the bubbling system seems not selective for A-T region at the parameters we have tested.

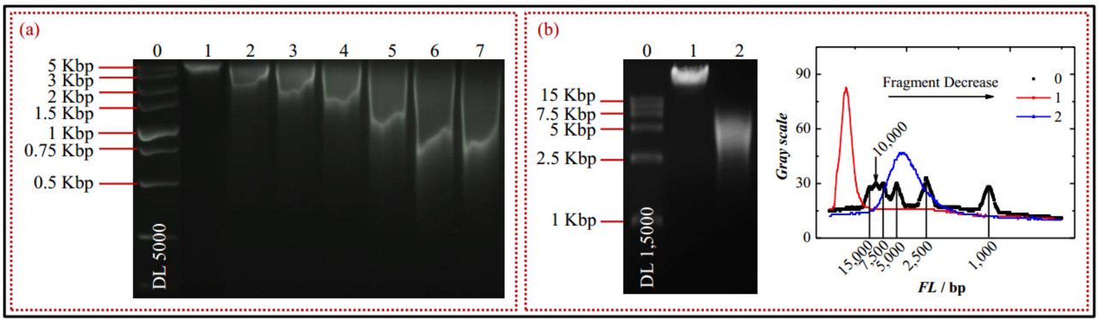

Figure S5. (a) Gel images of the DNAs fragmentation results of Herring Sperm DNA: 0 - marker (DL 5,000), 1 - untreated Herring Sperm DNA (positive control), 2, 3, 4, 5, 6 and 7 - fragmented lambda DNAs for 30 min at 0.05, 0.1, 0.15, 0.2, 0.25, 0.3 MPa, respectively. **(b) DNA fragmentation results of lambda DNAs:** 0 - marker (DL 15,000)，1 - untreated lambda DNA (positive control), 2 - fragmented lambda DNA for 30 min at 0.20 MPa.

DNA fragmentation with silicon oil on the top

Silicon oil was put on top of the DNA solution to investigate the effect of bubble bursting on the DNA fragmentation. 1.5 mL of 100 μg/mL DNA solutions with or without 1 mL silicon oil on the top of it were bubbled for 30 min at 0.10 MPa. The gel images of original DNA sample (No. 1), bubbling with oil coverage (No. 2) and without oil coverage (No. 3) were demonstrated in Fig. S6.

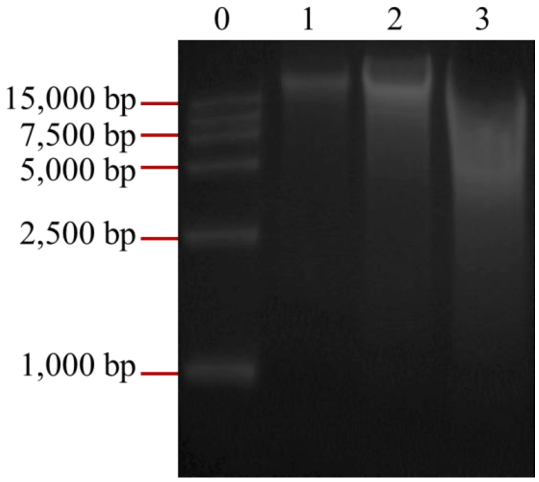

Figure S6. Gel images of DNA fragmentation results with or without silicon oil coverage: 0 - marker (DL 15,000), 1 - original genomic DNA sample (positive control), 2 - fragmented DNA sample with oil coverage (30 min at 0.10 MPa), and 3 - fragmented DNA sample without oil coverage (30 min at 0.10 MPa).
